# Characterizing microbiomes of African fermented foods in a global context

**DOI:** 10.1099/mic.0.001695

**Published:** 2026-05-05

**Authors:** John Leech, Yemisi D. Obafemi, Samuel Breselge, Taiwo Aremu, Adewale Olusegun Obadina, Martins Ebunoluwa Itohan, Chibundu N. Ezekiel, Charles Parkouda, Abel Tankoano, Korotimi Traoré, Kolawole Banwo, Angela Parry-Hanson Kunadu, Felix Kwashie Madilo, Abiodun I. Sanni, Omotade Richard Ogunremi, Gbemisola Onipede, Damaris Achieng Odeny, Cynthia Otieno, Marcus J. Claesson, Paul D. Cotter

**Affiliations:** 1 Teagasc Food Research Centre, Fermoy, Cork, Ireland; 2 Department of Biological Sciences, Covenant University, Ota, Ogun State, Nigeria; 3 APC Microbiome Ireland, University College Cork, Cork, Ireland; 4 VistaMilk, Cork, Ireland; 5 Department of Food Science and Technology, Federal University of Agriculture, Abeokuta, Nigeria; 6 Department of Biotechnology and Food Technology, Faculty of Science, Doornfontein Campus, University of Johannesburg, Johannesburg, South Africa; 7 Department of Agricultural Sciences, Institute for Bioanalytics and Agro-Metabolomics, BOKU University, Konrad Lorenz Str. 20, 3430 Tulln, Austria; 8 Department of Biological Sciences, Faculty of Science, Clifford University, Owerrinta, Ihie Campus, Abia State, Nigeria; 9 Department of Microbiology, Babcock University, Ilishan Remo, Ogun State, Nigeria; 10 CNRST/IRSAT/Department of Food Technology, Ouagadougou 03, Burkina Faso; 11 Department of Microbiology, University of Ibadan, Oyo State, Nigeria; 12 Department of Nutrition and Food Science, University of Ghana, Legon, Accra, Ghana; 13 Department of Food Science and Technology, Ho Technical University, Volta Region, Ghana; 14 Department of Microbiology and Biotechnology, Abiola Ajimobi Technical University, Ibadan, Nigeria; 15 Department of Microbiology, Federal University of Health Sciences, Ila Orangun, Nigeria; 16 The International Crops Research Institute for the Semi-Arid Tropics (ICRISAT) - Eastern and Southern Africa (ESA), United Nations Avenue, ICRAF Campus, Nairobi, Kenya; 17 School of Microbiology, University College Cork, Cork, Ireland

**Keywords:** antimicrobial resistance (AMR), beta diversity, fermented foods, metagenome-assembled genomes (MAGs), metagenomics, virulence

## Abstract

Fermentation plays a vital role globally, shaping traditional diets and enhancing food preservation, nutrition and flavour. With over 5,000 varieties of fermented foods globally, the microbiomes of many of these have yet to be explored, particularly with respect to those produced in some regions of Africa. To begin to address this knowledge gap, we conducted a shotgun metagenomics-based analysis of 91 fermented foods produced in Burkina Faso, Ghana, Kenya and Nigeria and compared them to a larger, global curated Food Metagenomic Database (cFMD). As for other studies of fermented food microbiomes in general, the substrate that was fermented emerged as the primary determinant of microbial beta diversity within the current African dataset and between the broader cFMD dataset. However, it was notable that the newly studied samples showed a small but statistically significant geographic signal. The African samples also displayed more alpha diversity than the global dataset, with cassava-, seed- and grain-based samples having the highest alpha diversity among the African foods. We also characterized the functional and antimicrobial profiles of all food-derived metagenome-assembled genomes (MAGs), noting the prevalence of pathways associated with carbohydrate metabolism across both African and non-African MAGs and an absence of known antimicrobial resistance genes in numerous genera. These findings not only expand our fundamental understanding of Africa’s under-studied fermented food microbiomes but also lay the foundation for starter culture development tailored to local substrates and conditions, fostering opportunities to enhance product safety, quality and scalability while retaining key characteristics associated with the original, artisanal product.

## Data Availability

The raw sequencing data generated in this study have been deposited in the NCBI Sequence Read Archive (SRA) under BioProject accession number PRJNA1052643. The specific SRA accession numbers are SRX23261875–SRX23261965.

## Introduction

Artisanal fermented foods have regained considerable popularity in western society due to the health benefits attributed to some such foods and a potential to positively impact the gastrointestinal microbiome [1,3]. Foods like kombucha, sourdough and kefir are among those receiving attention from both researchers and consumers [1,4,7]. However, many fermented foods from certain parts of the world remain relatively understudied and underappreciated outside of the region in which they are produced. Notably, Africa has historically produced a vast array of fermented foods that continue to be important parts of local diets. These foods are produced from a wide range of substrates, including grain, legumes, fruit, palm sap, cassava and milk [8,9].

Local African fermented products are generally regarded as a source of safe and affordable foods that can contribute to combating poverty and malnutrition. Fermentation can also be beneficial for the health of its consumers for several other reasons such as the pre-digestion of foods to make nutrients more bioavailable, removal of allergens, the potential for associated strains to have probiotic-associated properties, respectively, and in many instances, an abundance of potential prebiotics [10]. For some African consumers, fermentation plays an important role in food preservation, especially for those for whom refrigeration is not accessible. The production of fermented foods also provides opportunities for employment and economic growth through the commercialization of such foods. However, the majority of fermented foods in Africa are produced through spontaneous fermentation or backslopping methods, the latter involving a process whereby a portion of the previously fermented food is used to inoculate the next batch. These approaches can lead to fluctuations in quality and also provide challenges with respect to the potential for large-scale production [11]. To fully realize the economic potential of these foods for their regions and to make specific foods available to a larger population of consumers, the development of starter cultures can be an important step towards the production of products of consistent quality on an industrial scale. However, the development of such starters first requires an understanding of the species and strains that are present in the artisanal form of these products.

Despite the great need for such studies, investigations of the microbiomes of fermented foods produced within Africa continue to rely heavily on culture-based techniques, with a limited number of studies employing PCR/molecular [12,15] or sequencing-based approaches [16,17]. The knowledge gained from a greater understanding of the microbiomes of such foods could help to inform local fermentation industry processes, while optimizing the nutritional and health-promoting properties of these foods. In this study, we employed shotgun metagenomics to survey the microbiome of a broad variety of African fermented foods to better understand their microbial composition and associated functional profiles.

## Methods

### Sample collection

Ninety-one fermented food samples were collected from the various countries by our collaborating researchers. Samples, at the point of consumption or immediately prior to cooking, were collected in Genotek OMNI-gene tubes. The following metadata was collected for all samples: country of origin, substrate (main ingredient), solid or liquid, aerobic or semi-aerobic or anaerobic, backslopped or spontaneous. A total of 2.5 g of tube contents (food and buffer) was used for DNA extraction. The tube contents were centrifuged in 2 ml Eppendorf tubes (X2) for 10 min at 13,000 r.p.m. The supernatant was discarded, and the pellet was used for subsequent steps. The Powerfaecal extraction kit and protocol was used to extract DNA from the samples using a final 50 µl elution.

### Sequencing

Samples were then library prepped as per Illumina Nextera XT protocol (Illumina). DNA was quantified using a Qubit High Sensitivity dsDNA assay. Library quality was assessed on an Agilent High Sensitivity DNA chip, and quantification by quantitative PCR using the KAPA Library Quantification Kit for Illumina (Roche). Sequencing was carried out on the NextSeq 500 using a 300-cycle High Output v2 kit. Raw sequencing data have been deposited in the NCBI Sequence Read Archive under BioProject accession number PRJNA1052643.

### Bioinformatics analyses

Both the curated Food Metagenomic Database (cFMD) data and the African fermented food data all followed the following pipeline. The acquired sequences were quality trimmed with host removal using kneaddata v0.6.1. Kaiju v1.6.3 was used to assign taxonomy to the sequences using the NCBI non-redundant database including fungi and microbial eukaryotes [18]. Taxa with <0.1% relative abundance were removed due to the high likelihood of obtaining false positives. Contigs were produced from the fasta reads using ibda v1.1.337, which were subsequently binned using MetaBAT into metagenome-assembled genomes (MAGs) [19,20]. CheckM v1.0.18 was used to assess the quality of the MAGs [21]. MAGs were then filtered for quality using the following thresholds. Completeness >90% and contamination of <5% were classified as high-quality MAGs. Completeness of <90% but >80% and contamination of <5% were classified as medium quality, and finally, all other MAGs were deemed as low quality. FastANI was used to assign taxonomy to these MAGs, using the NCBI genome reference database as a reference [22].

The virulence factor (VF) nucleotide database was constructed using the core nucleotide sequences from the Virulence Factors Database (VFDB) [23]. The downloaded sequences were formatted into a blast nucleotide database using the ‘makeblastdb’ utility [24]. To identify potential VFs, sequences were subjected to blastn analysis against the prepared VF database. The blast searches were conducted with an *e*-value cutoff of 0.00001, a word size of 28 and a minimum percent identity of 60%.

To identify carbohydrate-active enzymes (CAZymes), we used dbCAN [25]. CAZyme annotation was performed using dbCAN v3.0.1 run_dbcan in prokaryotic mode. In the absence of user-specified thresholds, the pipeline applied the standard dbCAN defaults for its constituent tools, which include HMMER searches against dbCAN HMMs and dbCAN-sub-HMMs with an E-value cutoff of~1×10⁻¹⁵ and minimum model coverage of~35%, and DIAMOND searches against the CAZy database with an *E*-value cutoff of~1×10⁻¹⁰² (as documented for dbCAN).

Antimicrobial resistance (AMR) genes were identified using AMRFinder. Sequences were analysed with a minimum identity of 60% and coverage of 60% [26].

All statistical results were generated in R v4.0 using the vegan package, and plots were produced in R using ggplot2 [27,29].

## Results

### Diversity and key drivers in fermented foods

**Fig. 1.**
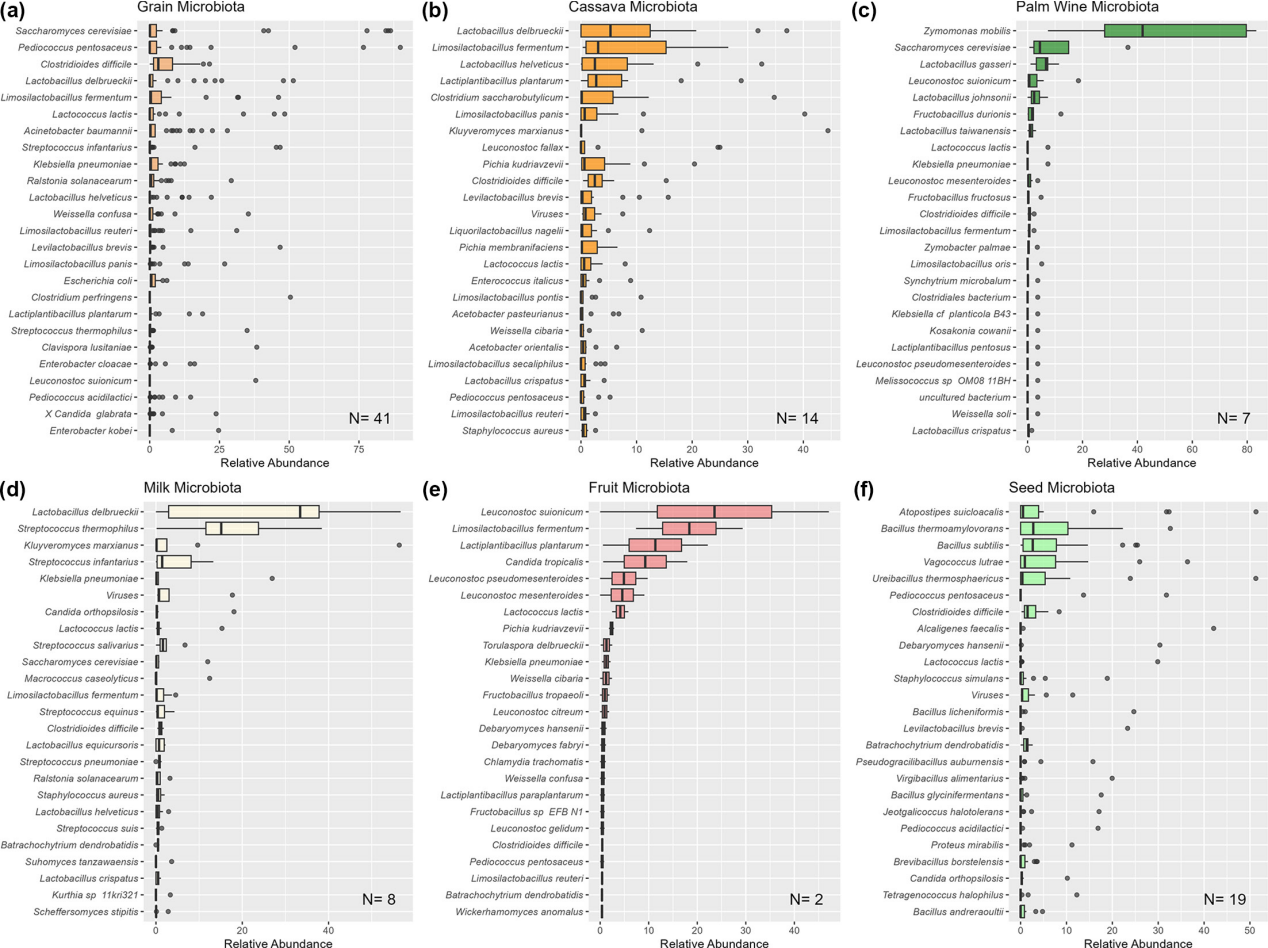
Boxplots showing the range of relative abundances for the top 25 species detected across each of the 6 main food type substrates.

Ninety-one fermented food samples were sourced from Nigeria (72), Ghana (9), Burkina Faso (6) and Kenya (4), representing 61 different types of fermented foods, and subjected to shotgun metagenomic sequencing. A full description of these samples can be found in Table 1. The samples were produced from a range of substrates including various grains (sorghum, millet, maize and guinea corn), various other plants (palm, locust bean, cassava and plantain) and cow’s milk. The samples were produced via spontaneous fermentation or backslopping and involved a variety of aerobic, semi-aerobic and anaerobic approaches. A total of 848 species were found at above 0.1% relative abundance across the 91 food samples. The top 25 most abundant species for each broad ingredient type are depicted in Fig. 1. Of these, *Lactobacillus delbrueckii* (5.9% of all assignments)*, Saccharomyces cerevisiae* (5.9%), *Limosilactobacillus fermentum* (3.9%), *Pediococcus pentosaceus* (3.8%) and *Zymomonas mobilis* (3.7%) were the most abundant taxa across all fermented food samples in the current study. *Clostridioides difficile*, notable for being a pathogen, was identified in all 6 broad ingredient types (Fig. 1).

**Table 1.** Description of samples included in this study The columns include study-specific sample identifier, name of food, country of origin, mode of fermentation initiation, mode of fermentation condition (aerobic, anaerobic, semi-anaerobic), solid or liquid food, type of ingredient used and more generic binning of food type.

Sample	Name	Country	Starter	Aerobic	Solid	Ingredient (broad)	Ingredient (specific)
AF01	Ogi-maize	Nigeria	Spontaneous	Semi-aerobic	Semi-solid	Grain	Grain
AF02	Ogi baba – millet	Nigeria	Spontaneous	Semi-aerobic	Semi-solid	Grain	Grain
AF03	Gari	Nigeria	Spontaneous	Aerobic	Solid	Cassava	Cassava
AF04	Fufu	Nigeria	Spontaneous	Aerobic	Solid	Cassava	Cassava
AF05	Kunun	Nigeria	Spontaneous	Aerobic	Liquid	Grain	Grain
AF06	Iru	Nigeria	Spontaneous	Aerobic	Solid	Seeds	Grain
AF07	Palm wine	Nigeria	Spontaneous	Aerobic	Liquid	Palm	Palm
AF08	Ogi/Koko Sorghum	Nigeria	Spontaneous	Semi-aerobic	Semi-solid	Grain	Grain
AF09	Ogiri	Nigeria	Spontaneous	Semi-aerobic	Solid	Seeds	Grain
AF10	Iru	Nigeria	Spontaneous	Aerobic	Solid	Seeds	Grain
AF11	Ugba	Nigeria	Spontaneous	Semi-aerobic	Solid	Seeds	Grain
AF12	Ogi	Nigeria	Spontaneous	Semi-aerobic	Semi-solid	Grain	Grain
AF14	Wara	Nigeria	Spontaneous	Aerobic	Solid	Milk	Milk
AF15	Ogiri	Nigeria	Spontaneous	Semi-aerobic	Solid	Seeds	Grain
AF16	Lafun	Nigeria	Spontaneous	Aerobic	Solid	Cassava	Cassava
AF17	Pito	Nigeria	Backslopped	Anaerobic	Liquid	Grain	Grain
AF18	Otika	Nigeria	Spontaneous	Aerobic	Liquid	Grain	Grain
AF19	Palm wine	Nigeria	Spontaneous	Aerobic	Liquid	Palm	Palm
AF20	Kunnun-zaki	Nigeria	Spontaneous	Aerobic	Liquid	Grain	Grain
AF21	Palm wine	Nigeria	Spontaneous	Aerobic	Liquid	Palm	Palm
AF22	Nono	Nigeria	Spontaneous	Aerobic	Liquid	Milk	Milk
AF23	Palm wine	Nigeria	Spontaneous	Aerobic	Liquid	Palm	Palm
AF24	Dawa Dawa	Ghana	Spontaneous	Anaerobic	Solid	Seeds	Grain
AF25	Kantong	Ghana	Spontaneous	Anaerobic	Solid	Seeds	Grain
AF26	Red millet Zankom	Ghana	Spontaneous	Anaerobic	Liquid	Grain	Grain
AF27	Wagashi	Ghana	Spontaneous	Anaerobic	Solid	Milk	Milk
AF28	Dagati Pito	Ghana	Backslopped	Anaerobic	Liquid	Grain	Grain
AF29	White Millet Zonkom	Ghana	Spontaneous	Anaerobic	Liquid	Grain	Grain
AF30	Brukina	Ghana	Spontaneous	Anaerobic	Liquid	Grain	Grain
AF31	Burukutu	Ghana	Spontaneous	Anaerobic	Solid	Grain	Grain
AF32	Frafra Pito	Ghana	Backslopped	Anaerobic	Liquid	Grain	Grain
AF33	Kunun-zaki	Nigeria	Spontaneous	Aerobic	Liquid	Grain	Grain
AF34	Nono	Nigeria	Spontaneous	Aerobic	Liquid	Milk	Milk
AF35	Ogi	Nigeria	Spontaneous	Semi-aerobic	Semi-solid	Grain	Grain
AF36	Maasa	Nigeria	Spontaneous	Semi-aerobic	Semi-solid	Grain	Grain
AF37	Ogi White	Nigeria	Spontaneous	Semi-aerobic	Semi-solid	Grain	Grain
AF38	Agadagidi	Nigeria	Spontaneous	Aerobic	Liquid	Fruit	Fruit
AF39	Emu	Nigeria	Spontaneous	Aerobic	Liquid	Palm	Palm
AF40	Burukutu	Nigeria	Spontaneous	Aerobic	Liquid	Grain	Grain
AF41	Otika	Nigeria	Spontaneous	Aerobic	Liquid	Grain	Grain
AF42	Wara	Nigeria	Spontaneous	Aerobic	Solid	Milk	Milk
AF43	Ugba	Nigeria	Spontaneous	Semi-aerobic	Solid	Seeds	Grain
AF44	Gari	Nigeria	Spontaneous	Aerobic	Solid	Cassava	Cassava
AF45	Ogiri	Nigeria	Spontaneous	Semi-aerobic	Solid	Cassava	Cassava
AF46	Ogi	Nigeria	Spontaneous	Semi-aerobic	Semi-solid	Grain	Grain
AF47	Okpheye	Nigeria	Spontaneous	Semi-aerobic	Solid	Seeds	Grain
AF48	Iru Pete	Nigeria	Spontaneous	Semi-aerobic	Solid	Seeds	Grain
AF49	Fura	Nigeria	Spontaneous	Aerobic	Solid	Grain	Grain
AF50	Ogi	Nigeria	Spontaneous	Semi-aerobic	Semi-solid	Grain	Grain
AF51	Fufu	Nigeria	Spontaneous	Aerobic	Solid	Cassava	Cassava
AF52	Iru Woro	Nigeria	Spontaneous	Semi-aerobic	Solid	Seeds	Grain
AF53	Lafun	Nigeria	Spontaneous	Aerobic	Solid	Cassava	Cassava
AF54	Gari	Nigeria	Spontaneous	Aerobic	Solid	Cassava	Cassava
AF55	Palm wine	Nigeria	Spontaneous	Aerobic	Liquid	Palm	Palm
AF56	Iru	Nigeria	Spontaneous	Aerobic	Solid	Seeds	Grain
AF57	Ogiri	Nigeria	Spontaneous	Aerobic	Semi-solid	Seeds	Grain
AF58	Okpehe	Nigeria	Backslopped	Aerobic	Solid	Seeds	Grain
AF59	Ugba	Nigeria	Spontaneous	Aerobic	Solid	Seeds	Grain
AF60	Ogi maize	Nigeria	Spontaneous	Aerobic	Solid	Grain	Grain
AF61	Ogi baba	Nigeria	Spontaneous	Aerobic	Solid	Grain	Grain
AF62	Koko	Nigeria	Spontaneous	Aerobic	Solid	Grain	Grain
AF63	Otiru	Nigeria	Spontaneous	Aerobic	Solid	Seeds	Grain
AF64	Nunu	Nigeria	Backslopped	Aerobic	Liquid	Milk	Milk
AF65	Wara	Nigeria	Spontaneous	Aerobic	Solid	Milk	Milk
AF66	Obiolor	Nigeria	Backslopped	Aerobic	Liquid	Grain	Grain
AF67	Burunkutu	Nigeria	Backslopped	Aerobic	Liquid	Grain	Grain
AF68	Sekete	Nigeria	Backslopped	Aerobic	Liquid	Grain	Grain
AF69	Gari	Nigeria	Spontaneous	Aerobic	Solid	Cassava	Cassava
AF70	Lafun	Nigeria	Spontaneous	Aerobic	Solid	Cassava	Cassava
AF71	Fufu	Nigeria	Spontaneous	Aerobic	Solid	Cassava	Cassava
AF72	Agadagidi	Nigeria	Spontaneous	Aerobic	Liquid	Fruit	Fruit
AF73	Ugi – finger millet	Kenya	Spontaneous	Anaerobic	Semi-solid	Grain	Grain
AF74	Ugi – sorghum and pearl millet	Kenya	Spontaneous	Anaerobic	Semi-solid	Grain	Grain
AF75	Ugi – finger millet and sorghum	Kenya	Spontaneous	Anaerobic	Semi-solid	Grain	Grain
AF76	Ugi – finger millet and pearl millet	Kenya	Spontaneous	Anaerobic	Semi-solid	Grain	Grain
AF77	Dolo	Burkina Faso	Backslopped	Semi-aerobic	Liquid	Grain	Grain
AF78	Massa	Burkina Faso	Spontaneous	Aerobic	Liquid	Grain	Grain
AF79	Foura	Burkina Faso	Spontaneous	Aerobic	Solid	Grain	Grain
AF80	Soumbala	Burkina Faso	Spontaneous	Aerobic	Solid	Seeds	Grain
AF81	Mou	Burkina Faso	Spontaneous	Aerobic	Solid	Cassava	Cassava
AF82	Attieke	Burkina Faso	Backslopped	Semi-aerobic	Solid	Cassava	Cassava
AF83	Iru	Nigeria	Spontaneous	Aerobic	Solid	Seeds	Grain
AF84	Garri	Nigeria	Spontaneous	Aerobic	Solid	Cassava	Cassava
AF86	Kunu – 3	Nigeria	Spontaneous	Aerobic	Liquid	Grain	Grain
AF87	Maize Ogi yellow	Nigeria	Spontaneous	Semi-aerobic	Semi-solid	Grain	Grain
AF88	Sorghum Ogi (ogi baba)	Nigeria	Spontaneous	Semi-aerobic	Semi-solid	Grain	Grain
AF89	Fufu	Nigeria	Spontaneous	Aerobic	Solid	Cassava	Cassava
AF91	Palm wine	Nigeria	Spontaneous	Aerobic	Liquid	Palm	Palm
AF92	Kunu – 2	Nigeria	Spontaneous	Aerobic	Liquid	Grain	Grain
AF93	Nono	Nigeria	Spontaneous	Aerobic	Liquid	Milk	Milk
AF94	Kunu – 1	Nigeria	Spontaneous	Aerobic	Liquid	Grain	Grain

The number of species detected above 0.1% relative abundance per sample ranged from 16 in palm wine to 138 in ugba, both of which were produced in Nigeria. The alpha and beta diversity of the fermented food microbiomes from the current study were compared against foods from a larger food database, the cFMD [30]. From this resource, we used 1,888 food metagenomes, spanning a wide range of matrices including dairy products (milk, whey, cheese, yoghurt and kefir), meat and sausage products, cereal- and grain-based foods and beverages (e.g. sourdough, beer and injera), legume- and vegetable-based ferments (e.g. soybean products, sauerkraut and pepper sauces) and sugary or plant-based beverages (e.g. honey ferments, pulque, tea, coffee, wine and water kefir). These represent mainly fermented foods, along with some pre- and post-fermented substrates, and do not include the additional food-production-environment samples that are also part of the wider cFMD database.

**Fig. 2.**
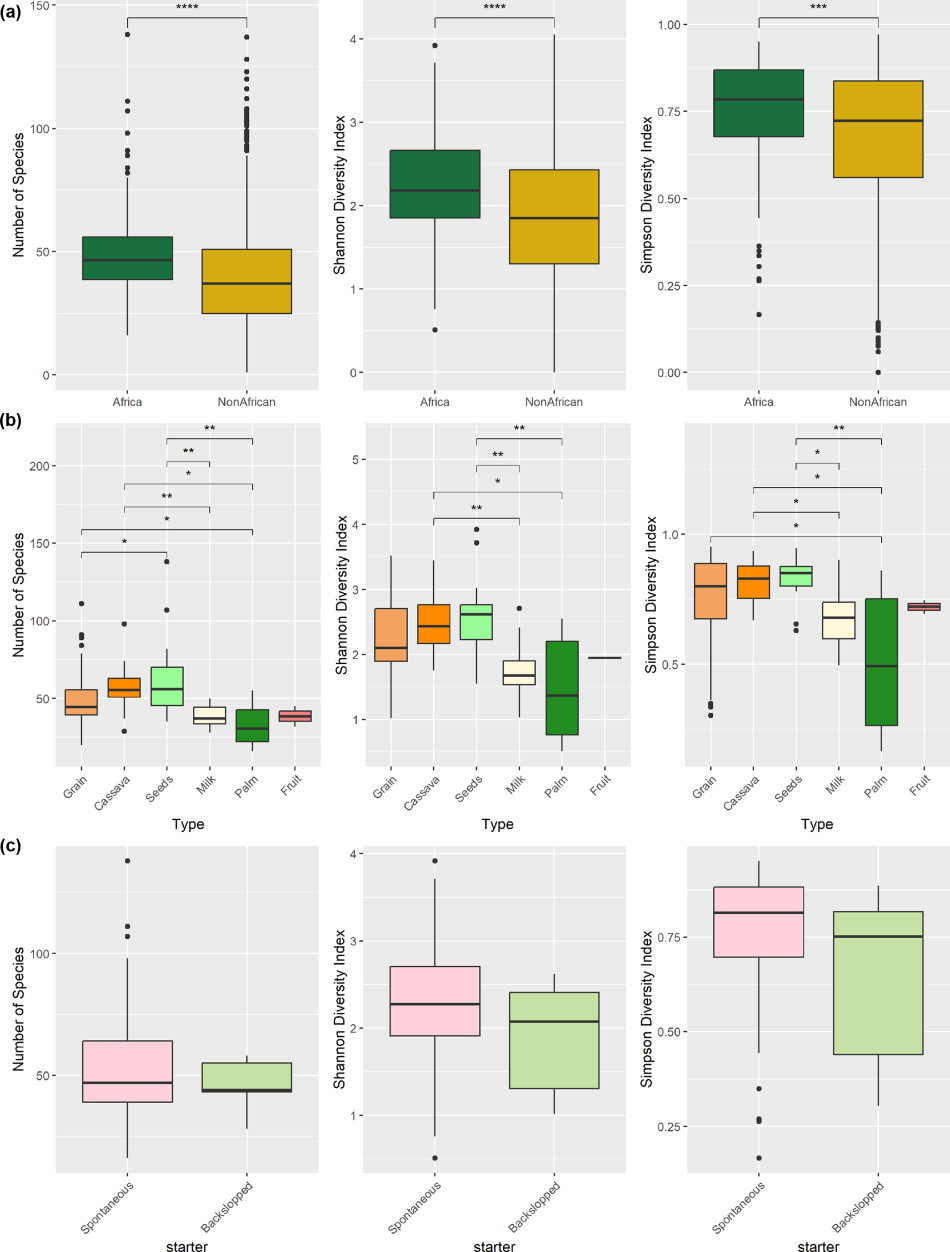
Integrated alpha diversity analysis comparing microbial communities from African versus non-African samples and exploring diversity within African food fermentations. (**a**) Boxplots for species richness, Shannon diversity and Simpson diversity indices for samples from both the current study and the cFMD dataset, grouped as ‘Africa’ or ‘NonAfrican’. (b) Diversity metrics across the African food types (grain, cassava, palm, milk, fruit and seeds). (c) African samples by fermentation starter type (spontaneous vs. backslopped). Statistical significance between groups is indicated by asterisks (*), where one or more stars denote significant differences (e.g. *P<0.05, **P<0.01, ***P<0.001 and ****P<0.0001) as determined by t-tests (for species richness) and Wilcoxon tests (for Shannon and Simpson indices).

Looking at the foods by origin, African samples had significantly higher alpha diversity than the non-African global samples (Fig. 2a). Within the African dataset, grain-, cassava- and seed-based fermented samples had significantly higher alpha diversity than samples made from milk and palm sap. The diversity of fruit-derived samples was also low, but the number of samples available precluded a statistical comparison (Fig. 2b). Alpha diversity was also examined across African fermented samples based on whether they were backslopped (inoculated using a portion of a previous successful fermentation) or spontaneously fermented (Fig. 2c). While spontaneous fermentations had higher alpha diversity scores across all three metrics used, i.e., number of species, Shannon’s diversity index and Simpson’s diversity, these differences were not significant.

**Fig. 3.**
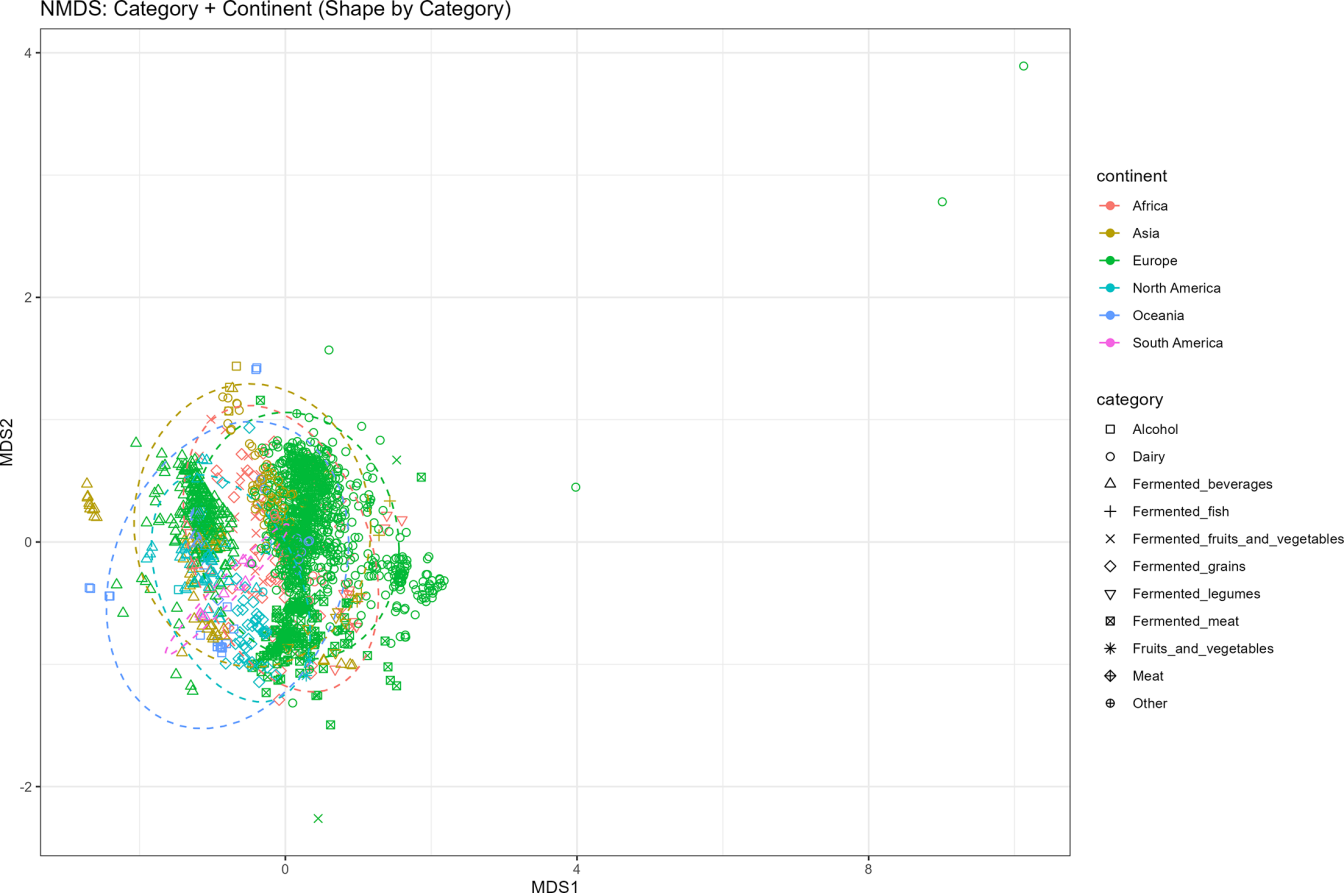
NMDS of Bray–Curtis distances between the 91 African samples and the 1,888 global samples from the cFMD dataset, calculated for the species-level composition. Samples are coloured by continent, while the shapes represent the main fermented ingredient. Both Continent, African versus non-African samples and substrate type were significantly different (Adonis, *P*<0.001, *P*<0.001 and *P*<0.001, respectively).

For beta diversity analyses, the combined dataset was examined from two perspectives. Firstly, permutational multivariate ANOVA (PERMANOVA) (Adonis) was carried out on the samples using food type (i.e. based on substrate used) and continent as factors (Fig. 3, Table 2). Together, these factors explained ~32.08% of the total variation, with type being the most influential (*R*^2^=0.3078, *F*=9.44, *P*=0.001). This was followed by continent (*R*^2^=0.0129, *F*=7.36, *P*=0.001). The remaining 65.56% of the variation was unexplained, suggesting that additional unmeasured factors influence microbial community composition. To specifically examine if the African fermented samples’ microbiomes were particularly different from other global samples, given the uniqueness of many of the African fermented samples and preparation methods, samples were classified as either African or non-African, and PERMANOVA was repeated using food type and African vs. non-African as factors (Table 3). This analysis explained 32.58% of the variation, again with type as the dominant factor (*R*^2^=0.3258, *F*=9.83, *P*=0.001), followed by African vs. non-African (*R*^2^=0.00058, *F*=1.63, *P*=0.03). The remaining 66.80% of the variation was unexplained. While most metadata was not available across all fermented food datasets, beta diversity was examined between starter culture samples and samples produced via spontaneous fermentation. This analysis was conducted on both the combined African dataset and a previous global fermented food study [5] and on the African dataset on its own. The combined analysis explained 1.89% of the variation, again with type as the dominant factor (*R*^2^=0.0193, *F*=2.88, *P*=0.001) and the African only dataset (*R*^2^=0.0189, *F*=1.71, *P*=0.023), revealing that there are small but significant differences between fermented samples based on whether or not starter cultures/backslopping was used. These results highlight that food type is the dominant driver of microbial community composition in fermented samples, with geographic origin (continent or African classification) also playing a role, though to a lesser extent. Within the full dataset, three cheese-brine samples (GL_Q03-22, GL_Q04-22 and GL_Q06-22) were clear non-metric multidimensional scaling (NMDS) outliers, driven by unusually high dominance of halophilic *Chromohalobacter* species, a pattern not observed in the other cheese-brine or fermented food samples.

**Table 2.** PERMANOVA/Adonis results showing the effects of type (food substrate) and continent on microbial community composition Columns include degrees of freedom (Df), sum of squares (SumOfSqs), proportion of variance explained (*R*^2^), pseudo-F statistic (*F*) and the associated *P*-value (Pr(>*F*)).

Term	Df	SumOfSqs	*R* ^2^	*F*	Pr(>*F*)
Type	93	262.5492	0.307794	9.439584	0.001
Continent	5	11.00074	0.012896	7.356596	0.001
Residual	1,870	559.2636	0.65564	na	na
Total	1,968	853.0035	1	na	na

**Table 3.** PERMANOVA/Adonis results showing the effects of type (food substrate) and African versus non-African origin on microbial community composition Columns include degrees of freedom (Df), sum of squares (SumOfSqs), proportion of variance explained (*R*^2^), pseudo-F statistic (*F*) and the associated *P*-value (Pr(>*F*)).

Term	Df	SumOfSqs	*R* ^2^	*F*	Pr(>*F*)
Type	93	277.9083	0.3258	9.828527	0.001
Africa_vs_rest	1	0.494144	0.000579	1.625261	0.03
Residual	1,874	569.7702	0.667958	na	na
Total	1,968	853.0035	1	na	na

Beta diversity was assessed within the African dataset using Bray–Curtis dissimilarity, visualized through NMDS ordination and statistically tested using PERMANOVA (Fig. S1 and Table S1, available in the online Supplementary Material). Both Type and Country significantly influenced community composition. Type explained 22.4% of the variance (*R*² = 0.2239, *F*=4.86, *P*=0.001), making it the strongest driver of differences among samples. Country also had a significant but smaller effect, explaining 6.0% of the variance (*R*² = 0.0599, *F*=2.17, *P*=0.001). The remaining variation (71.8%) was attributable to residual factors not captured by these variables. NMDS plots showed clear separation by Type, with more subtle structuring by Country.

### Taxonomic description of individual fermented food types

#### Fermented grains

The largest group of samples investigated in this study were those generated using grains as substrates (41), with a range of cereal ingredients including maize, millet, sorghum and guinea corn (Fig. 1). Of these, samples from millet-based fermentations were most numerous (28). *S. cerevisiae* was the most abundant species across all grain samples, and it is likely that repeated backslopping (in many cases) contributed to its dominance. *P. pentosaceus* was the next most abundant taxa, with another *Pediococcus* species, *Pediococcus acidilactici*, also appearing among the most abundant. Lactic acid bacteria (LAB) were also very common, with *L. delbrueckii*, *Lm. fermentum*, *Streptococcus infantarius* and *Lactobacillus helveticus* being well represented.

#### Cassava ferments

Fourteen food samples were produced through the fermentation of cassava. Attieke was produced via backslopping, while all other cassava samples were produced via spontaneous fermentation. All cassava ferments were solid-state fermentations. *L. delbreuckii* was the most abundant species and, along with *Lm. fermentum*, was present in all samples of gari. LAB made up the majority of the most abundant genera found in cassava ferments, with *Lactobacillus*, *Limosilactobacillus*, *Lactiplantibacillus*, *Leuconostoc*, *Levilactobacillus*, *Liquorilactobacillus*, *Pediococcus*, *Weisella* and *Lactococcus* all well represented. Acetic acid bacteria (AAB) were frequently found in cassava samples also, with *Acetobacter pasteurianus* and *Acetobacter orientalis* appearing in the top 25 most abundant species in cassava microbiomes. Yeasts such as *Pichia kudriavzevii*, *Pichia membranifaciens* and *Kluyveromyces marxianus* were the most abundant eukaryotic microbes found in cassava-based fermented samples.

#### Palm wine/Emu

There were seven palm wine samples included in the study, all from Nigeria. All palm wine samples were aerobically, spontaneously produced beverages. *Z. mobilis* was the most abundant species found in these samples, followed by *S. cerevisiae*. LAB were well represented in palm wine fermentations with *Lactobacillus gasseri*, *Leuconostoc suionicum*, *Lactobacillus johnsonii* and *Fructobacillus durionis* being among the most abundant. Palm wine samples were notable for the occurrence of *Fructobacillus* in general. Other genera of LAB appeared in the top 25 taxa, with *Lactiplantibacillus*, *Weissella* and *Limosilactobacillus* all being identified.

#### Fermented milk-based foods

Four milk-based fermented samples, nunu, nono, wagashi and wara, were studied. Samples were generally spontaneously and aerobically fermented, with the exception of nunu, which was backslopped, and wagashi, which was produced anaerobically. Milk samples included both solid and liquid foods. *L. delbrueckii*, *Streptococcus thermophilus*, *K. marxianus* and *St. infantarius* were the most abundant taxa in these samples. As for many of the other food substrates, the fermented milk microbiota was dominated by LAB species. Nunu, a food that was the focus of a previous food-safety metagenomic study [11], was found to have *L. delbrueckii* (37%), *St. thermophilus* (23%), *S. cerevisiae* (12%), *Lm. fermentum* (3.7%), *Streptococcus salivarius* (2.4%), *L. helveticus* (1.6%), *Acetobacter cibinongensis* (1.3%), *Staphylococcus epidermidis* (1.3%), *C. difficile* (1.2%) and *Streptococcus pneumoniae* (0.9%).

#### Fruit-based fermented foods

Agadagidi, a beverage produced via the spontaneous, aerobic fermentation of plantain, was included in the study. Its composition differed across samples, being either dominated by *Lc. suionicum* or *Lm. fermentum*. *Lactiplantibacillus plantarum*, *Candida tropicalis*, *Leuconostoc pseudomesenteroides*, *Leuconostoc mesenteroides* and *Lactococcus lactis* contributed to the majority of the other species detected.

#### Seed-based fermented foods

Melon seed, locust bean and oil seed were among the substrates that contributed to seed-based fermented foods. The microbiome of these samples differed most considerably from the other African samples by virtue of containing a wider range of microbes and being less dominated by LAB. While LAB and yeast were among the most abundant taxa, five species of *Bacillus* were among the top 25 taxa. *Bacillus thermoamylovorans* was the most abundant of these *Bacillus* and was the second most abundant seed microbe after *Atopostipes suicloacalis. Bacillus subtilis* was the third most abundant taxa. Among the represented LAB taxa, *Vagococcus lutrae*, *P. pentosaceus* and *La. lactis* were among the next most abundant taxa. *Debaryomyces hansenii* was the most abundant yeast in the seed fermentations.

### VFs, antimicrobial resistance genes and CAZyme profiles reveal global distribution of functional genes

**Fig. 4.**
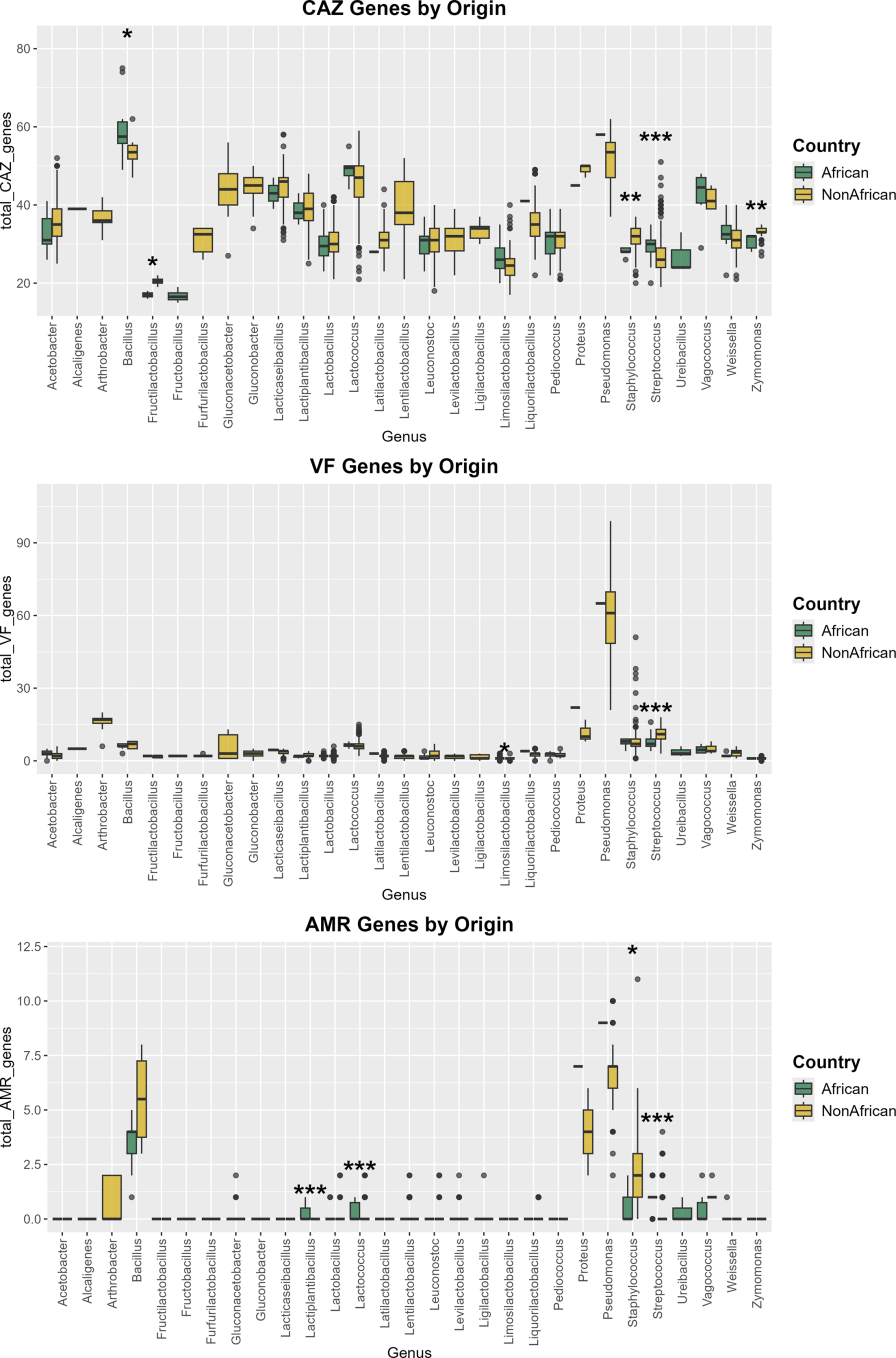
Box plots of (from top to bottom) VFs, AMR genes and CAZyme genes found in African and non-African fermented food MAGs. A large global dataset, comprising over 3,901 MAGs, was used for these analyses. Three levels of significant differences are shown: *P*<0.05 (*), *P*<0.01 (**) and *P*<0.001 (***).

Using a large dataset of 3,901 MAGs, 180 from the current study and 3,721 from cFMD collection [30], we compared the abundance of CAZymes, VFs and antimicrobial resistance (AMR) genes between MAGs originating from African and non-African environments (Fig. 4). Altogether, these 3,901 MAGs encompassed 29 genera, providing a broad basis for assessing geographical differences in functional potential. Among these, the genera with the largest representation overall were *Lactococcus* (6 African MAGs and 643 non-African MAGs), *Leuconostoc* (10 African MAGs and 399 non-African MAGs) and *Lacticaseibacillus* (2 African MAGs and 379 non-African MAGs). Among African-derived MAGs, the most prevalent were from the genera *Limosilactobacillus* (36 African MAGs and 32 non-African MAGs), *Lactobacillus* (28 African MAGs and 258 non-African MAGs) and *Streptococcus* (25 African MAGs and 258 non-African MAGs). These genera accounted for a significant proportion of the dataset and contributed substantially to the observed trends. Statistical comparisons between African and non-African MAGs were conducted using the Wilcoxon rank-sum test, with adjusted *P*-values calculated to account for multiple comparisons. For the pairwise comparisons below, differences were considered significant at adjusted *P*-values of *P*<0.05, and three levels of significance were reported: *P*<0.05 (*), *P*<0.01 (**) and *P*<0.001 (***).

#### CAZyme gene content

The abundance of CAZyme genes, which are crucial for carbohydrate metabolism, varied substantially across genera. Genera with the highest CAZyme gene abundance included *Bacillus* (African mean: 59.7, non-African mean: 53.8), *Pseudomonas* (African mean: 58.0, non-African mean: 51.7) and *Lactococcus* (African mean: 49.2, non-African mean: 45.5). In contrast, genera such as *Fructilactobacillus* (African mean: 17.0, non-African mean: 20.4) and *Limosilactobacillus* (African mean: 26.4, non-African mean: 25.7) exhibited lower CAZyme gene counts.

Several genera showed significant differences in CAZyme gene abundance between African and non-African MAGs. *Streptococcus*, a highly represented genus, had significantly higher CAZyme gene counts in African MAGs (mean: 29.3) compared to non-African MAGs (mean: 27.4, *P*<0.001). Similarly, *Zymomonas* exhibited higher CAZyme gene counts in non-African MAGs (mean: 33.2) compared to African MAGs (mean: 30.6, *P*<0.01). A significant difference was also observed in *Staphylococcus*, where non-African MAGs had higher counts (mean: 31.4) compared to African MAGs (mean: 28.0, *P*<0.01). Smaller but significant differences were noted for *Fructilactobacillus* and *Bacillus* (*P*<0.05).

The frequency of each detected CAZyme, coloured by CAZyme family, is illustrated in Fig. S2.

#### VF-like gene content

While the core VF database contains experimentally validated VFs, it is important to recognize that many of these genes may have additional functions and do not necessarily contribute exclusively to virulence. As a result, their presence in our analysis does not inherently indicate a pathogenic role but rather highlights factors related to genes associated with virulence under at least some circumstances. The abundance of VF-like genes varied considerably across the dataset according to genera. Genera with the highest mean VF gene counts included *Pseudomonas* (African mean: 65.0, non-African mean: 59.0) and *Proteus* (African mean: 22.0, non-African mean: 11.7). *Pseudomonas* accounted for 47 MAGs, comprising 13 known species and 2 unknown species, including a single African MAG, identified as *Pseudomonas fluorescens*, from a Nigerian sample of gari. In contrast, *Zymomonas* (African mean: 1.0, non-African mean: 1.1) and *Limosilactobacillus* (African mean: 1.17, non-African mean: 0.88) consistently exhibited the lowest VF-like gene counts.

Significant differences were observed in two key genera. Non-African *Streptococcus* MAGs had a markedly greater VF-like gene count (mean: 10.9) than those of African origin (mean: 7.7, *P*<0.001). Additionally*, Limosilactobacillus* showed slightly higher VF-like counts in African MAGs (mean: 1.17) compared to non-African MAGs (mean: 0.88, *P*<0.05).

These findings indicate significant geographic variation in VF profiles, with non-African MAGs, particularly of *Streptococcus*, demonstrating higher pathogenic potential. Such differences may reflect adaptations to environmental or host-associated factors. Positive VF-like hits were detected in *Lactococcus* (African mean: 6.5, non-African mean: 6.4) and *Lacticaseibacillus* (African mean: 4.5, non-African mean: 3.5). In *Lactococcus* MAGs, the most frequently detected VF-associated genes included phosphopyruvate hydratase (enolase), glyceraldehyde-3-phosphate dehydrogenase (GAPDH) and trigger factors, each present in over 50% of the MAGs. In *Lacticaseibacillus* MAGs, positive VF hits included genes encoding Hsp60 (HtpB), phosphopyruvate hydratase (enolase), Clp ATP-binding endopeptidase (ClpC) and endocarditis-specific antigen (EfaA). As noted above, the *Lactococcus* and *Lacticaseibacillus*-associated genes are not thought to be true VF but rather resemble stress resistance and other genes that contribute to the fitness of pathogenic bacteria.

#### AMR gene content

The distribution of AMR genes across the dataset was highly variable, with some genera having substantial counts while others exhibited little to no representation of these genes. Genera with the highest AMR gene abundance included *Proteus* (African mean: 7.0, non-African mean: 4.0) and *Pseudomonas* (African mean: 9.0, non-African mean: 6.7). Conversely, no AMR genes were detected in either African or non-African MAGs from the genera *Zymomonas*, *Acetobacter*, *Latilactobacillus*, *Lacticaseibacillus*, *Pediococcus*, *Fructilactobacillus*, *Limosilactobacillus* and *Fructobacillus*.

Significant differences in AMR gene abundance were observed for several genera. *Streptococcus*, one of the most represented genera, had significantly higher AMR gene counts in African MAGs (mean: 1.0) compared to non-African MAGs (mean: 0.29, *P*<0.001). A similar pattern was observed in *Lactiplantibacillus* and *Lactococcus*, where African MAGs showed higher AMR counts (mean: 0.33; mean: 0.33) compared to non-African MAGs (mean: 0.0, *P*<0.001; mean: 0.04, *P*<0.001, respectively). In contrast, *Staphylococcus* MAGs had higher AMR counts in non-African assemblies (mean: 1.76) than the African equivalents (mean: 0.60, *P*<0.05).

## Discussion

### Microbial diversity in African fermented foods

This article describes a shotgun metagenomics-based analysis of a diverse array of African fermented foods. Our results reveal that the primary driver of microbial diversity in these samples and the broader global dataset is the substrate. The investigation covered 91 fermented food samples from Nigeria, Ghana, Burkina Faso and Kenya and 1,888 global fermented foods (including pre- and post-fermented substrates), uncovering a broad spectrum of microbial communities and functional profiles.

Geography, at both the continental scale and when comparing African to non-African samples, was statistically significant, though the effect size was very small in both datasets. Cultural fermentation practices within Africa and the unique nature (including unique substrates) of the fermented foods produced are the most likely cause of these differences. The African samples consisted of a large quantity of grain, cassava and seed fermentations, which were less prevalent outside of Africa in general. Within the African dataset, beta diversity analyses showed the same pattern: substrate (i.e. food type) was again the primary driver of community differences, while country had a smaller but statistically significant effect. This mirrors observations from other fermented-food studies, where substrate consistently shapes microbiota, whereas geographic effects are more variable and often non-significant.

Consistent with previous research on fermented foods from other regions, the type of substrate was the most significant factor influencing the microbial diversity of the fermented samples. This aligns with findings from studies on European and Asian fermented foods, where the substrate’s nutritional composition and physicochemical properties were found to shape the microbial community structure [5,30]. While the use of starter cultures or backslopping also drove beta diversity between the African foods and in another fermented food dataset, the effect size was very small. It is important to note that the African dataset is unevenly sampled, with most samples originating from Nigeria and only four countries represented, which should be considered when interpreting geographical patterns.

Grain-, cassava- and seed-based samples exhibited the highest microbial diversity. Grain-based fermentations were dominated by *S. cerevisiae* and several species of LAB, including *Pediococcus. Streptococcus* also featured regularly within grain fermentations, with *St. infantarius* and *St. thermophilus* ranking highly among the most abundant taxa, consistent with previous studies [31,32]. While *St. thermophilus* is a species to which many starter cultures belong, this is not the case for *St. infantarius*, though this study highlights potential for commercial purposes. However, there are some safety concerns relating to *St. infantarius* and more in-depth safety evaluations of specific strains would be required before being adopted by fermented food producers as starters [33].

Consistent with previous culture-based studies, cassava-based ferments, predominantly from Nigeria and Burkina Faso, were characterized by a high abundance of *L. delbrueckii*, *Lm. fermentum* and other LAB [14]. AAB such as *A. pasteurianus* are also present at high abundance. The dominance of these bacteria is likely due to their ability to thrive in the acidic and carbohydrate-rich environment provided by cassava. Yeasts are represented in these samples by *K. marxianus* and different species of *Pichia*, such as *Pc. kudriavzevii* and *Pc. membranifaciens. Pichia* and *Kluyveromyces* are receiving more attention from researchers and fermented food producers in recent times due to *Pichia’s* limited alcohol production and *Kluyveromyces’* ability to grow in high temperature, fast growth and utilization of a wide array of substrates [34,35].

The seed fermentations were among the most interesting by virtue of the extent to which the taxonomy of the associated microbiome differed from that of other samples. *At. suicloacalis* was the most abundant microbe in these samples. *Atopostipes* is a lesser-known genus of LAB and was first isolated from pig slurry pits [36]. *Atopostipes* has been investigated for its biotechnological applications including waste management and valorization [37]. *Bacillus* featured heavily among the seed ferments. *Bacillus* are known for their proteolysis and are commonly found in other high protein substrates elsewhere in the world, such as legume fermentations [38]. *Bacillus* are also receiving much attention as potential probiotics, with strains of *Bacillus coagulans*, recently renamed *Heyndrickxia coagulans*, receiving most attention [39]. Some species of *Bacillus* also come with safety concerns, and the *Bacillus cereus* complexes are well-known enterotoxin producers [40]. However, all of the *Bacillus* uncovered here are better known for their involvement in food fermentations. These *Bacillus* have potential amylolytic and proteolytic capabilities of great importance to industrial applications and food fermentations [38].

Palm wine fermentations, among the least diverse samples in the current study, were either dominated by *Z. mobilis* or *S. cerevisiae,* but most of the less abundant adjuncts were LAB [41]. *L. gasseri* and *Lc. suionicum* were common in these samples, potentially highlighting LAB capable of persisting in foods with higher alcohol content. *Fructobacillus* also featured in these samples and is well known for its ability to convert fructose to lactic acid [42]. Consistent with previous studies, fermented milk samples had low alpha diversity compared to other samples [5,30]. *L. delbrueckii* and *St. thermophilus* were the most highly abundant milk-associated species within the study. These are also the most commonly used species for yoghurt fermentations around the world. *K. marxianus* and *La. lactis* also featured quite regularly. These species are also commonly found in dairy fermentations, such as milk kefir, globally [43,44]. Unsurprisingly for milk fermentations, several LAB featured among the most abundant taxa. Although there were only two samples of fruit fermentations, both produced via the fermentation of plantain, they exhibited a unique combination of LAB, including some *Fructobacillus*, and various yeasts such as *Pichia*, *Torulaspora* and *Debaryomyces*. While fruit is commonly added to flavour commercial fermented beverages, such as kombucha and water kefir, they are not as frequently fermented as substrates themselves. These plantain microbiota could be a good starting point for exploring non-alcoholic fruit fermentations for a wider diversity of fruits. *C. difficile* appeared frequently in the dataset, reflecting its widespread presence in soil and raw agricultural materials. As a highly resilient spore-former, it can survive harvesting, handling and processing steps. Its detection in fermented foods therefore most likely represents persistence of environmental spores rather than in-product growth. Low-abundance detections of other putative pathogens, such as those found in nunu samples in the current study, should also be interpreted cautiously. In previous work on nunu, metagenomic pathogen signatures could not be recovered by culture [11], illustrating that such hits may reflect misclassification or non-viable DNA rather than true contamination.

The comparative analysis of MAGs from African and non-African regions reveals significant differences in functional gene content, particularly in CAZ, VFs and AMR genes. These differences are reflective of both ecological niches and the specific roles that certain bacterial genera play within their environments, especially with regard to metabolic functions like fermentation and their adaptation to external pressures such as antimicrobial use. One of the most striking findings in this study is the prominence of *Bacillus* and *Pseudomonas* across both African and non-African MAGs in terms of CAZyme gene content. These genera consistently displayed the highest levels of CAZyme genes, underscoring their capacity to degrade complex carbohydrates. LAB and AAB also showed substantial CAZyme gene content, reflecting their importance in fermentation processes. The high CAZyme gene content in LAB genera such as *Lactococcus* and *Lacticaseibacillus* across both African and non-African MAGs highlights their critical role in fermentative breakdown of carbohydrates, which is central to food production and preservation processes globally. Similarly, AAB genera like *Acetobacter* and *Gluconacetobacter* demonstrated consistent contributions to carbohydrate metabolism, particularly in non-African MAGs. These findings reinforce the global importance of LAB and AAB genera in traditional and industrial fermentation processes.

In comparing the CAZyme gene repertoires of microbial MAGs sourced from African and non-African fermented foods, several notable patterns emerged. *Streptococcus*, one of the most abundant genera in many fermentations, displayed significantly higher CAZyme gene counts in African MAGs, pointing to a possible adaptation (or domestication) to the specific carbohydrates or substrates prevalent in non-African fermentations where *St. thermophilus* is regularly used as a starter culture [45]. Conversely, *Zymomonas* and *Staphylococcus* MAGs from non-African contexts showed higher CAZyme gene abundances, which may reflect the influence of different raw materials or fermentation techniques more common outside Africa. Even the smaller yet significant differences observed in *Fructilactobacillus* and *Bacillus* underscore how regional ecologies and cultural practices can shape microbial functional capacities. Such variations in CAZyme gene content are especially important given the pivotal role these enzymes play in breaking down complex carbohydrates, impacting both fermentation efficiency and the sensory attributes of the final product. These findings align with broader evidence that microbial genomic traits often mirror the local environments and substrates where they evolve or are cultivated [45,48]. Future work that links these genomic differences to specific functional outcomes, for instance, flavour compound formation, fermentation kinetics or probiotic potential, could further clarify how region-specific conditions influence the microbial genomics of fermented foods.

While *Bacillus* and *Pseudomonas* exhibited high metabolic capacities, they also displayed significant differences in virulence potential. *Pseudomonas* consistently showed the highest VF gene counts in both African and non-African MAGs, making it a genus of considerable concern due to its potential pathogenicity. The high VF gene content in *Pseudomonas* across both regions highlights its potential role as an opportunistic pathogen. When examining VF gene distributions across African and non-African strains, *Streptococcus* emerged as a key genus exhibiting higher VF gene counts in non-African MAGs (mean: 10.9) than in African MAGs (mean: 7.7, *P*<0.001). In some African contexts, certain *Streptococcus* species, such as *S. infantarius*, are known to function as relatively benign or even food-grade microbes, particularly in dairy fermentations. The lower VF content observed among African *Streptococcus* MAGs might reflect this shift towards more commensal or beneficial roles, as opposed to the potentially greater pathogenic potential implied by higher VF counts in non-African MAGs. In contrast, LAB and AAB genera consistently exhibited low VF-like gene counts, consistent with the generally regarded as safe status of many associated taxa. This is particularly significant given their widespread use in food fermentation. The detection of putative VF genes in *Lactococcus* and *Lacticaseibacillus*, including enolase, GAPDH, trigger factors, Hsp60 (HtpB), Clp ATP-binding endopeptidase (ClpC) and endocarditis-specific antigen (EfaA), underscores how core cellular functions can overlap with pathogenic mechanisms in certain bacterial contexts. For instance, enolase and GAPDH are integral to glycolysis but are also known to facilitate bacterial adhesion and immune evasion in pathogenic species [49,50]. Likewise, trigger factors and Hsp60 (HtpB) are crucial for proper protein folding and stress response, but in some pathogenic bacteria, they may also mediate host–pathogen interactions, enhancing bacterial survival in host tissues [51,52]. ClpC, similarly, functions in proteolysis and stress adaptation yet can contribute to virulence if it modulates host immune responses [53,54]. Finally, EfaA (endocarditis-specific antigen), while closely associated with endocarditis in pathogenic strains, may serve more benign roles in adhesion or niche colonization for food-related LAB [55].

Taken together, these gene detections do not indicate harmful potential in the context of fermented foods. Rather, they highlight the broad physiological utility of these genes, ranging from basic metabolic processes to stress tolerance and, in some cases, adhesion. Given the relatively permissive thresholds applied for VF and AMR detection (60% identity and 60% coverage), these results should be interpreted with caution, as such thresholds can increase the likelihood of false positives; however, they were chosen to ensure adequate sensitivity when screening diverse MAGs from multiple regions. This overlap suggests that commonly used VF databases can flag genetic elements that are essential for bacterial survival but are not strictly ‘virulence’ traits. AMR is a critical issue in both environmental and clinical contexts, and this study provides insight into the distribution of AMR genes across different bacterial genera in African and non-African regions. *Pseudomonas* again stood out, exhibiting the highest AMR gene counts. This suggests that *Pseudomonas* may be exposed to higher levels of antimicrobial pressures, possibly due to agricultural or clinical factors. The presence of AMR genes in *Bacillus* across both regions further highlights the adaptability of this genus to environments where antimicrobials are present, although the levels were lower compared to *Pseudomonas*. The low AMR gene content in LAB and AAB genera is noteworthy. These bacteria, which are crucial for fermentation, exhibited minimal resistance across both African and non-African MAGs, reinforcing their role in environments where antimicrobial pressures are likely minimal, such as controlled fermentation processes. This makes them reliable candidates for use in food production, where the absence of AMR genes is highly desirable to avoid the transmission of resistance traits to pathogens. Notable geographic variations emerged in AMR gene abundance across several key bacterial genera. *Streptococcus*, one of the most prevalent genera in these datasets, showed substantially higher AMR gene counts in African MAGs (mean: 1.0) compared to non-African MAGs (mean: 0.29, *P*<0.001), suggesting region-specific selective pressures or adaptation. A similar pattern was observed in *Lactiplantibacillus* (mean: 0.33 vs. 0.0, *P*<0.001) and *Lactococcus* (mean: 0.33 vs. 0.04, *P*<0.001), where African MAGs consistently carried more putative AMR genes. In contrast, *Staphylococcus* displayed higher AMR counts in non-African MAGs (mean: 1.76) than in African MAGs (mean: 0.60, *P*<0.05). These differences may reflect local antibiotic use, environmental influences or horizontal gene transfer events shaping the resistome in different regions. Additionally, these findings may hint at contrasting antibiotic use patterns in clinical (*Staphylococcus* in non-African) versus agricultural settings across different regions, although direct evidence linking these MAG data to specific practices remains limited. Although the detection of AMR genes in food-associated bacteria raises important questions regarding safety and public health, further studies integrating phenotypic assays are essential to clarify whether these genes are active, clinically relevant or simply part of the broader genetic repertoire of microbes involved in fermentation [56]. The consistent presence of LAB and AAB genera across both African and non-African MAGs emphasizes their critical roles in fermentation. The high CAZyme gene content in these genera aligns with their known functions in breaking down complex carbohydrates during fermentation, whether in traditional African fermented foods or in traditional and industrial processes in non-African regions. The low VF and AMR gene content in these genera across regions further supports their safety and utility in food production. These findings have important implications for the use of LAB and AAB genera in fermentation, particularly in ensuring the production of safe, high-quality food products.

This study provides a foundational understanding of the microbial diversity and functional potential of African fermented foods. The findings highlight the critical role of substrate and fermentation conditions in shaping microbial communities. The information from the detailed functional profiles offers exciting opportunities for future research and the development of new fermentation technologies. By enhancing our understanding of these traditional foods, we can improve their nutritional value, safety and economic potential, contributing to food security and public health in the regions where they are produced. 

## Supplementary material

10.1099/mic.0.001695 Supplementary Material 1.

## Abbreviations

AAB: acetic acid bacteria
AMR: antimicrobial resistance
cFMD: curated Food Metagenomic Database
GAPDH: glyceraldehyde-3-phosphate dehydrogenase
LAB: lactic acid bacteria
MAG: metagenome-assembled genome
NMDS: non-metric multidimensional scaling
PERMANOVA: permutational multivariate ANOVA
VF: virulence factor

## References

[1] Marco ML, Heeney D, Binda S, Cifelli CJ, Cotter PD (2017). Health benefits of fermented foods: microbiota and beyond. Curr Opin Biotechnol.

[2] Pasolli E, De Filippis F, Mauriello IE, Cumbo F, Walsh AM (2020). Large-scale genome-wide analysis links lactic acid bacteria from food with the gut microbiome. Nat Commun.

[3] Wastyk HC, Fragiadakis GK, Perelman D, Dahan D, Merrill BD (2021). Gut-microbiota-targeted diets modulate human immune status. Cell.

[4] Landis EA, Oliverio AM, McKenney EA, Nichols LM, Kfoury N (2021). The diversity and function of sourdough starter microbiomes. Elife.

[5] Leech J, Cabrera-Rubio R, Walsh AM, Macori G, Walsh CJ (2020). Fermented-food metagenomics reveals substrate-associated differences in taxonomy and health-associated and antibiotic resistance determinants. mSystems.

[6] Tamang JP, Watanabe K, Holzapfel WH (2016). Review: diversity of microorganisms in global fermented foods and beverages. Front Microbiol.

[7] Villarreal-Soto SA, Bouajila J, Pace M, Leech J, Cotter PD (2020). Metabolome-microbiome signatures in the fermented beverage, Kombucha. Int J Food Microbiol.

[8] Agyei D, Owusu-Kwarteng J, Akabanda F, Akomea-Frempong S (2020). Indigenous African fermented dairy products: processing technology, microbiology and health benefits. Crit Rev Food Sci Nutr.

[9] Obafemi YD, Oranusi SU, Ajanaku KO, Akinduti PA, Leech J (2022). African fermented foods: overview, emerging benefits, and novel approaches to microbiome profiling. *NPJ Sci Food*.

[10] Mukherjee A, Breselge S, Dimidi E, Marco ML, Cotter PD (2024). Fermented foods and gastrointestinal health: underlying mechanisms. Nat Rev Gastroenterol Hepatol.

[11] Walsh AM, Crispie F, Daari K, O’Sullivan O, Martin JC (2017). Strain-level metagenomic analysis of the fermented dairy beverage nunu highlights potential food safety risks. Appl Environ Microbiol.

[12] Adesulu-Dahunsi AT, Sanni AI, Jeyaram K, Banwo K (2017). Genetic diversity of *Lactobacillus plantarum* strains from some indigenous fermented foods in Nigeria. *LWT - Food Science and Technology*.

[13] Dakwa S, Sakyi-Dawson E, Diako C, Annan NT, Amoa-Awua WK (2005). Effect of boiling and roasting on the fermentation of soybeans into dawadawa (soy-dawadawa). Int J Food Microbiol.

[14] Wilfrid Padonou S, Nielsen DS, Hounhouigan JD, Thorsen L, Nago MC (2009). The microbiota of Lafun, an African traditional cassava food product. Int J Food Microbiol.

[15] Pswarayi F, Gänzle MG (2019). Composition and origin of the fermentation microbiota of mahewu, a Zimbabwean fermented cereal beverage. Appl Environ Microbiol.

[16] Bationo F, Seyoum Y, Chochois V, Tamene A, Kariluoto S (2023). Bacterial diversity and community structure of some traditional African and European cereal-based fermented foods identified by high-throughput sequencing. Food Biosci.

[17] Diaz M, Kellingray L, Akinyemi N, Adefiranye OO, Olaonipekun AB (2019). Comparison of the microbial composition of African fermented foods using amplicon sequencing. Sci Rep.

[18] Menzel P, Ng KL, Krogh A (2016). Fast and sensitive taxonomic classification for metagenomics with Kaiju. Nat Commun.

[19] Kang DD, Froula J, Egan R, Wang Z (2015). MetaBAT, an efficient tool for accurately reconstructing single genomes from complex microbial communities. PeerJ.

[20] Peng Y, Leung HCM, Yiu SM, Chin FYL (2012). IDBA-UD: a de novo assembler for single-cell and metagenomic sequencing data with highly uneven depth. Bioinformatics.

[21] Parks DH, Imelfort M, Skennerton CT, Hugenholtz P, Tyson GW (2015). CheckM: assessing the quality of microbial genomes recovered from isolates, single cells, and metagenomes. Genome Res.

[22] Jain C, Rodriguez-R LM, Phillippy AM, Konstantinidis KT, Aluru S (2018). High throughput ANI analysis of 90K prokaryotic genomes reveals clear species boundaries. Nat Commun.

[23] Chen L, Yang J, Yu J, Yao Z, Sun L (2005). VFDB: a reference database for bacterial virulence factors. Nucleic Acids Res.

[24] Camacho C, Coulouris G, Avagyan V, Ma N, Papadopoulos J (2009). BLAST+: architecture and applications. BMC Bioinformatics.

[25] Yin Y, Mao X, Yang J, Chen X, Mao F (2012). dbCAN: a web resource for automated carbohydrate-active enzyme annotation. Nucleic Acids Res.

[26] Feldgarden M, Brover V, Haft DH, Prasad AB, Slotta DJ (2019). Validating the AMRFinder tool and resistance gene database by using antimicrobial resistance genotype-phenotype correlations in a collection of isolates. Antimicrob Agents Chemother.

[27] Ihaka R, Gentleman R (1996). R: a language for data analysis and graphics. J Comput Graph Stat.

[28] Oksanen J, Kindt R, Legendre P, O’Hara B, Stevens MHH (2007). The vegan package. Community Ecology Package.

[29] Wickham H (2011). ggplot2. Wiley Interdisciplinary Reviews: Computational Statistics.

[30] Carlino N, Blanco-Míguez A, Punčochář M, Mengoni C, Pinto F (2024). Unexplored microbial diversity from 2,500 food metagenomes and links with the human microbiome. Cell.

[31] Blandino A, Al-Aseeri ME, Pandiella SS, Cantero D, Webb C (2003). Cereal-based fermented foods and beverages. Food Res Int.

[32] Nout MJR (2009). Rich nutrition from the poorest - cereal fermentations in Africa and Asia. Food Microbiol.

[33] Dos Santos KMO, de Matos CR, Salles HO, de Melo Franco BDG, Arellano K (2020). Exploring beneficial/virulence properties of two dairy-related strains of *Streptococcus infantarius* subsp. *infantarius*. Probiotics Antimicrob Proteins.

[34] Lane MM, Morrissey JP (2010). Kluyveromyces marxianus: a yeast emerging from its sister’s shadow. Fungal Biol Rev.

[35] Vicente J, Calderón F, Santos A, Marquina D, Benito S (2021). High potential of *Pichia kluyveri* and other *Pichia* species in wine technology. *IJMS*.

[36] Cotta MA, Whitehead TR, Collins MD, Lawson PA (2004). *Atopostipes suicloacale* gen. nov., sp. nov., isolated from an underground swine manure storage pit. Anaerobe.

[37] Guan R, Yuan H, Zhang L, Zuo X, Li X (2021). Combined pretreatment using CaO and liquid fraction of digestate of rice straw: Anaerobic digestion performance and electron transfer. Chin J Chem Eng.

[38] Li Z, Zheng M, Zheng J, Gänzle MG (2023). Bacillus species in food fermentations: an underappreciated group of organisms for safe use in food fermentations. Curr Opin Food Sci.

[39] Cutting SM (2011). Bacillus probiotics. Food Microbiol.

[40] Ehling-Schulz M, Fricker M, Scherer S (2004). Identification of emetic toxin producing *Bacillus cereus* strains by a novel molecular assay. FEMS Microbiol Lett.

[41] Chandrasekhar K, Sreevani S, Seshapani P, Pramodhakumari J (2012). A review on palm wine. Int J Res Biol Sci.

[42] Endo A, Maeno S, Tanizawa Y, Kneifel W, Arita M (2018). Fructophilic lactic acid bacteria, a unique group of fructose-fermenting microbes. Appl Environ Microbiol.

[43] Walsh AM, Crispie F, Kilcawley K, O’Sullivan O, O’Sullivan MG (2016). Microbial succession and flavor production in the fermented dairy beverage kefir. mSystems.

[44] Walsh LH, Coakley M, Walsh AM, Crispie F, O’Toole PW (2023). Analysis of the milk kefir pan-metagenome reveals four community types, core species, and associated metabolic pathways. iScience.

[45] Somerville V, Thierer N, Schmidt RS, Roetschi A, Braillard L (2024). Genomic and phenotypic imprints of microbial domestication on cheese starter cultures. Nat Commun.

[46] Gibbons JG, Rinker DC (2015). The genomics of microbial domestication in the fermented food environment. Curr Opin Genet Dev.

[47] Martiny JBH, Bohannan BJM, Brown JH, Colwell RK, Fuhrman JA (2006). Microbial biogeography: putting microorganisms on the map. Nat Rev Microbiol.

[48] Steensels J, Gallone B, Voordeckers K, Verstrepen KJ (2019). Domestication of industrial microbes. Curr Biol.

[49] Madureira P, Baptista M, Vieira M, Magalhães V, Camelo A (2007). Streptococcus agalactiae GAPDH is a virulence-associated immunomodulatory protein. J Immunol.

[50] Sha J, Erova TE, Alyea RA, Wang S, Olano JP (2009). Surface-expressed enolase contributes to the pathogenesis of clinical isolate SSU of *Aeromonas hydrophila*. J Bacteriol.

[51] Agashe VR, Guha S, Chang H-C, Genevaux P, Hayer-Hartl M (2004). Function of trigger factor and DnaK in multidomain protein folding: increase in yield at the expense of folding speed. Cell.

[52] Petit MA, Bedale W, Osipiuk J, Lu C, Rajagopalan M (1994). Sequential folding of UmuC by the Hsp70 and Hsp60 chaperone complexes of *Escherichia coli*. J Biol Chem.

[53] Ingmer H, Vogensen FK, Hammer K, Kilstrup M (1999). Disruption and analysis of the clpB, clpC, and clpE genes in *Lactococcus lactis*: ClpE, a new Clp family in gram-positive bacteria. J Bacteriol.

[54] Rouquette C, de Chastellier C, Nair S, Berche P (1998). The ClpC ATPase of *Listeria monocytogenes* is a general stress protein required for virulence and promoting early bacterial escape from the phagosome of macrophages. Mol Microbiol.

[55] Low YL, Jakubovics NS, Flatman JC, Jenkinson HF, Smith AW (2003). Manganese-dependent regulation of the endocarditis-associated virulence factor EfaA of *Enterococcus faecalis*. J Med Microbiol.

[56] Salamandane A, Leech J, Almeida R, Silva C, Crispie F (2024). Metagenomic analysis of the bacterial microbiome, resistome and virulome distinguishes Portuguese Serra da Estrela PDO cheeses from similar non-PDO cheeses: an exploratory approach. Food Res Int.

