# Supplementary material for: Characterizing microbiomes of African fermented foods in a global context
Source: Microbiology (Reading). 2026 May 5;172(5):001695. doi: 10.1099/mic.0.001695 (PMC13142294; doi:10.1099/mic.0.001695)
Supplement: Supplementary Material 1. [file mic-172-01695-s001.pdf]

Supplementary Materials

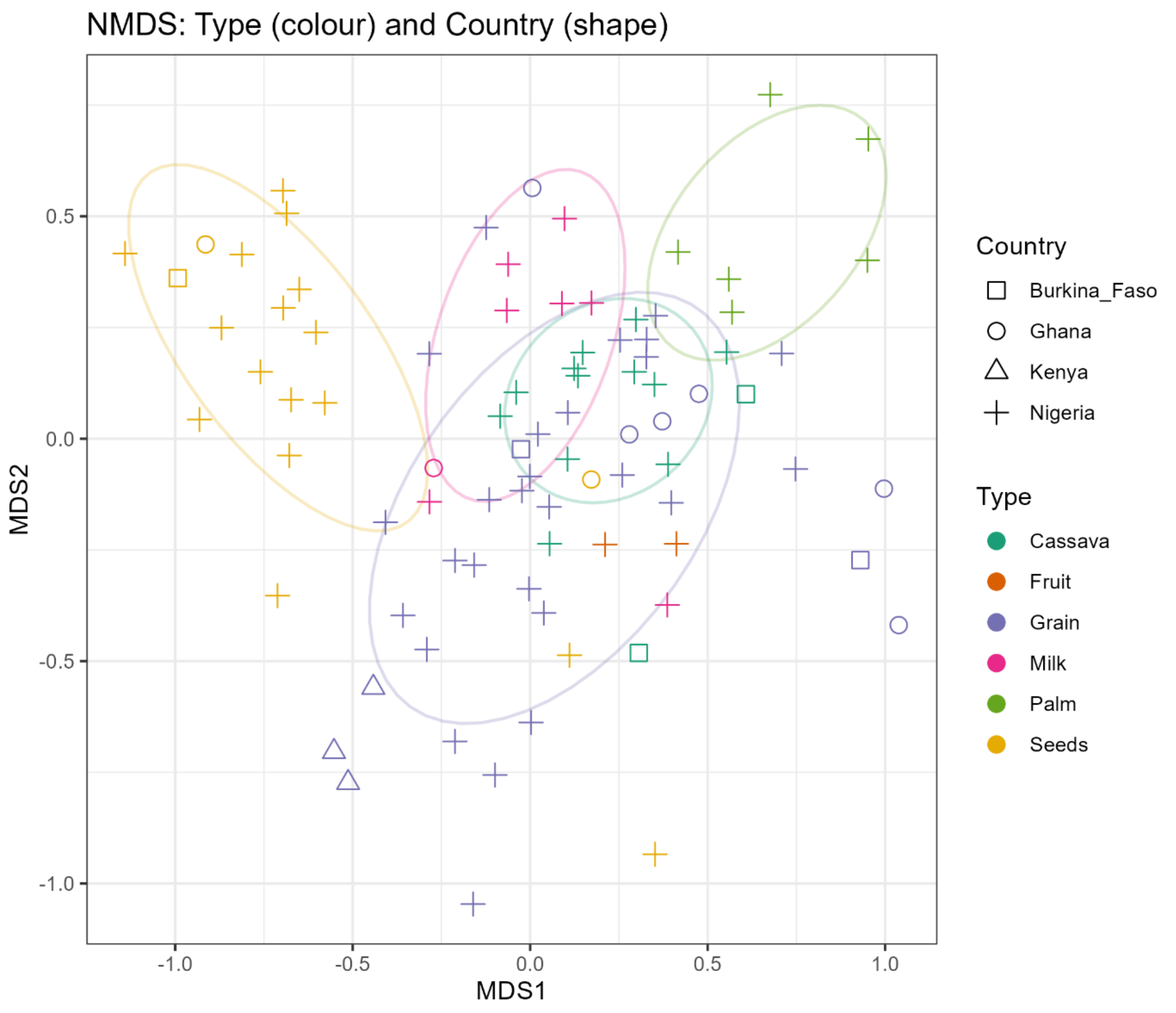

Supplementary Figure 1. Non-metric Multidimensional Scaling (NMDS) of Bray–Curtis distances for the 91 African food samples at the species level. Points are coloured by food type and shaped by country of origin. Both country and food type were significantly associated with community composition (Adonis, $p < 0.001$ for both).

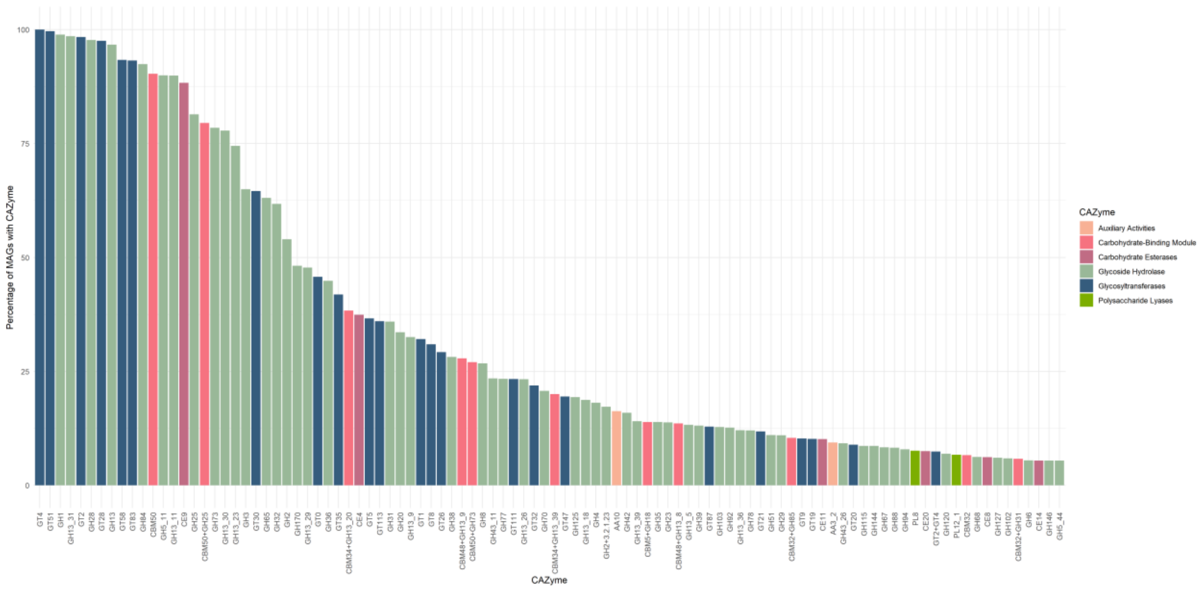

Supplementary Figure 2. Prevalence of CAZyme types across all food samples analysed (91 African samples and 1,888 global cFMD samples). The x-axis shows CAZyme types, and the y-axis indicates the percentage of samples in which each CAZyme was detected. Bars are coloured by CAZy family.

Supplementary Table 1

Term	Df	SumOfSqs	R2	F	Pr(>F)
type	5	8.445455	0.223874	4.862088	0.001
Country	3	2.260106	0.059911	2.168589	0.001
Residual	78	27.09723	0.7183	NA	NA
Total	86	37.72412	1	NA	NA

Supplementary Table 1: Permutational multivariate analysis of variance (PERMANOVA)/Adonis results showing the effects of type (food substrate), and Country on microbial community composition within the African dataset only. Columns include degrees of freedom (Df), sum of squares (SumOfSqs), proportion of variance explained (R2), pseudo-F statistic (F), and the associated p-value (Pr(>F)).
